# Observational Analysis of Point-of-Care Lactate Plus™ Meter in Preclinical Trauma Models

**DOI:** 10.3390/diagnostics14232641

**Published:** 2024-11-23

**Authors:** Catharina Gaeth, Jamila Duarte, Alvaro Rodriguez, Amber Powers, Randolph Stone

**Affiliations:** Combat Wound Care, United States Army Institute of Surgical Research, 3698 Chambers Pass, JBSA Fort Sam Houston, Houston, TX 78234, USA; catharina.c.gaeth.fm@health.mil (C.G.); jamila.m.duarte.civ@health.mil (J.D.); alvaro.v.rodriguez.mil@health.mil (A.R.); amber.m.powers2.ctr@health.mil (A.P.)

**Keywords:** point of care, lactate, prehospital, preclinical trauma models, swine, whole blood

## Abstract

Background/Objectives: Blood lactate concentration is often used to assess systemic hypoperfusion, tissue hypoxia, and sepsis in trauma patients and serves as a prognostic indicator and marker of response to therapy. Point-of-care (POC) devices provide rapid lactate measurements with a single drop of blood. In this study, lactate values from whole blood, measured with two POC devices, Abbott i-STAT^®^ and the Nova Biomedical Lactate (LA) Plus™ meter, are compared. Methods: An observational analysis of 760 blood samples from four preclinical trauma animal models was performed. Lactate was measured simultaneously with two POC devices (i-STAT^®^ and the Lactate Plus™ meter). The evaluation in the form of a Bland–Altman diagram showed the agreement of the tests. Results: The Spearman correlation was 0.9180 (95% CI 0.9056 to 0.9288) for i-STAT^®^ vs. the LA Plus™ meter. Both tests showed a significant increase in lactate after injury (*p* < 0.05). The i-STAT^®^ showed a small bias compared to the LA Plus™ meter (−0.0227 ± 0.4542 with 95% limits of agreement from −0.09129 to 0.8675). The LA Plus™ meter showed slightly higher values overall (0.0227 mmol/L), but the i-STAT^®^ showed higher results at lactate levels above 2.5 mmol/L. Conclusions: The observed similarity in performance between the two devices suggests that both are capable of reliably measuring lactate levels in a trauma situation. The LA Plus™ meter provides fast results with only one drop of blood. This study supports the prehospital use of POC devices.

## 1. Introduction

In prehospital and intensive care settings, the accurate and timely assessment of a patient’s metabolism is essential for guiding treatment and predicting outcomes. Lactate is a biomarker that has received considerable attention in this context and can be used as an indirect measure of anaerobic metabolism [1]. The body produces lactate during tissue hypoperfusion and hypoxia to meet its energy needs with anaerobic glycolysis [2]. Hyperlactatemia is characterized by elevated lactate levels and can lead to lactic acidosis. This condition is of particular concern in polytraumatized patients, such as those with hemorrhagic shock, where a decreased blood volume impairs the oxygen-carrying capacity, resulting in increased lactate production [3]. Elevated lactate concentrations can therefore be an indicator of the severity of shock and a predictor of the patient’s prognosis [4,5,6,7,8,9]. In addition, lactate levels are crucial for assessing the effectiveness of resuscitation measures [10,11,12,13,14,15].

However, lactate measurement is often dependent on centralized laboratory analysis, which can be time consuming and is not available in the prehospital setting. This could delay important therapeutic interventions [6]. Point-of-care (POC) diagnostics have proven to be valuable tools in the prehospital environment by acquiring additional information before a patient reaches the hospital. POC lactate measurements enable real-time assessment with minimal blood volume and are therefore essential for the rapid detection of metabolic changes prior to changes in vital signs such as heart rate and blood pressure.

The i-STAT^®^ CG4+ (Abbott Rapid Dx North America, LLC, Orlando, FL, USA) and the Lactate (LA) Plus™ meter (Nova Biomedical Corporation, Waltham, MA, USA) are two POC devices that can measure lactate in whole blood. Both devices use an electrochemical lactate oxidase biosensor to determine the lactate concentration in the blood sample [16,17].

In this study, we compared the two POC lactate measurement methods in four preclinical animal trauma models. Our primary objective was to evaluate the accuracy and utility of the LA Plus™ meter, a POC device, in comparison to the FDA-cleared i-STAT^®^ device [18,19]. By examining the correlation between these devices in porcine trauma models, we aimed to determine the accuracy of the LA Plus™ meter for use in prehospital and trauma care.

## 2. Materials and Methods

### 2.1. Animals

Research was conducted in compliance with the Animal Welfare Act, the implementing Animal Welfare Regulations, and the principles of the Guide for the Care and Use of Laboratory Animals. The facility’s Institutional Animal Care and Use Committee approved all research conducted in this study (burn and lung or skin inoculation: A-20-009, approved 11 December 2019; fecal peritonitis (FP): A-20-033, approved 23 September 2020; abdominal trauma (AT): A-22-009, approved 29 April 2022; and complex extremity trauma injury (CETI): A-22-019, approved 9 November 2022). The facility where this research was conducted is fully accredited by AAALAC International. Fifty-one female Yorkshire swine (Midwest Research Swine, Gibbon, MN, USA) and twenty-three male Sinclair swine (Sinclair Bio Resources, Auxvasse, MO, USA) were utilized in this study. Prior to experimentation, the swine were acclimated to the facilities with full access to food and water for a minimum of seven days.

### 2.2. Preclinical Trauma Model Timeline and General Procedures

A minimum of three days prior to experimental injury in anesthetized animals with adequate analgesia, catheters were placed in both an artery and vein (either femoral or jugular/carotid veins were used) to obtain blood for POC devices, CBC, and biochemistry laboratory panels. A telemetry implant device (Data Sciences International, New Brighton, MN, USA) was placed into the contralateral femoral artery to collect continuous basic vitals. The animals were allowed to recover after these procedures in their pen.

On the experimental injury day, one of four injuries were given to each animal while under anesthesia with adequate analgesia. For the FP model (*N* = 11), autologous feces were collected from the cage, diluted with saline, and warmed prior to the experiment to prevent hypothermia. A midline abdominal incision was made, and a drainage tube was placed to administer the fecal mixture. After administration, the tube was removed, and the skin was closed with sutures. For the AT model (*N* = 25), a midline abdominal incision was made, biopsy punches were placed in the small and large intestines, feces was expressed through holes from punches, mixed with saline, and administered. Then, the skin was closed in a similar manner to the FP model. For the burn model (*N* = 15), burns of 40% total body surface area burn were created on the back and flanks. Pseudomonas aeruginosa (PA) was then either administered via the lungs or applied to the burns. The complex extremity trauma injury (CETI) model (*N* = 23) consisted of extremity polytrauma with the removal of skin, muscle, and a small bone defect, 40% hemorrhage, and 2 h torniquet application.

A swine jacket was applied to each animal, and recovery was allowed after these procedures in their pen. The POC devices were used at blood collection time points before the injury and every 8–12 h after injury on conscious animals in their pen. All animals were preemptively given sustained-release buprenorphine analgesia and additional pain management as needed after instrumentation and injury. Depending on the model, up to 156 h after injury, surviving animals underwent anesthesia and euthanasia using a commercial solution after the final blood draw.

### 2.3. Blood Draws and Data Compilation

Blood was collected via arterial catheters placed on the instrumentation day. Lines were kept patent throughout the study period using a heplock locking solution and routine flushing of the lines. If the arterial line was no longer patent, then the venous line was used to draw whole blood. Data points were only included if the lactate levels were collected for the two lactate tests: i-STAT^®^ handheld blood analyzer (Abbott Rapid Dx North America, LLC, Orlando, FL, USA) and the LA Plus™ meter (Nova Biomedical Corporation, Waltham, MA, USA). Individual blood draws were excluded for time points during which 1 or more assays were not performed. The most common issue was the lack of availability of LA Plus™ meter strips or CG4+ cartridges for the i-STAT^®^ handheld blood analyzer when ordering was often delayed during the COVID-19 epidemic. To acquire the values in blood, for the i-STAT^®^ handheld blood analyzer, a CG4+ cold-stored cartridge was loaded with a blood sample (100 µL) and inserted into the device; meanwhile, for the LA Plus™ meter, a drop of blood (1 µL) was applied to the testing strip and inserted into the device for measurement. The i-STAT^®^ and LA Plus™ meter tests were performed immediately after each blood draw.

### 2.4. Statistical Analysis

Values were evaluated for the lactate test and combined to determine the trends across the preclinical trauma models. No outliers were removed from the study. The Bland–Altman analysis was utilized to compare the lactate tests to determine the level of agreement [20]. This analysis is a simple way to evaluate the bias between two measurements by determining their mean difference and constructing 95% agreement intervals of bias and standard deviation (SD). A statistical analysis was performed in GraphPad Prism 10.2.1 (GraphPad Software, Boston, MA, USA) using nonparametric Spearman correlation, linear progression, a Wilcox test, and a mixed-effect model comparing the tests at each time point with Tukey’s multiple comparison post-test. Significant differences were noted when *p* < 0.05.

## 3. Results

### 3.1. Preclinical Trauma Models Included in the Observational Study

This observational study combined the data from four preclinical trauma models in which the timeline was similar. The experimental timeline consisted of placing venous and arterial catheters approximately 3 days prior to the actual injury. Routine blood draws were collected before, immediately after the injury, and every 8–12 h after the injury until euthanasia. Table 1 indicates the preclinical trauma models, the number of animals for each, and the total blood draws that were included in this observational study. A total of 760 complete datasets were available from various time points of the four preclinical trauma models representing 94.4% (*N* = 760/805) of all possible blood draws. The most common reason for missing time points was a shortage of supplies.

### 3.2. Lactate Device Comparison over Time

A summary of the combined lactate values over time is shown in Table 2. To create this table, blood draws were sorted into time point categories. Any blood draw prior to injury (which comprised one to three draws for each animal) was placed in the ‘Before Injury’ group. The ‘Immediately after Injury’ group included blood draws once the animals had recovered from anesthesia, was back in its pen and conscious, and these only occurred once per animal. Then, each day included up to three blood draws, since they were performed every 8 h (for example, Day 1 consisted of the 8, 16, and 24 h time point). No significant differences (*p* < 0.05) in lactate results were observed (see Table 2) when comparing i-STAT^®^ and the LA Plus™ meter at all time points among the four different injury models. However, significant differences (*p* < 0.05) in lactate were observed (represented by # in Table 2) for each test comparing the sample collected ‘Immediately After Injury’ to all other time points for that particular test.

**Figure 1:**
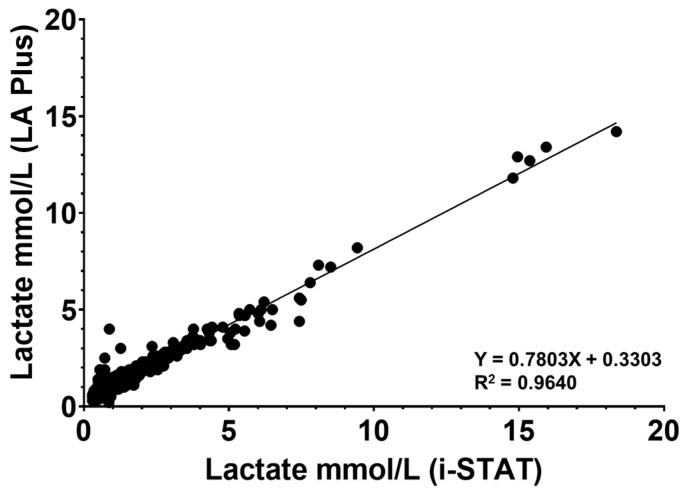
Linear regression comparing the LA Plus™ meter to i-STAT^®^ lactate device values from all the blood samples. Each corresponding lactate measurement from a blood draw are plotted on their respective axis. The equation for the trend line and associated goodness of fit or R^2^ is shown for the comparison. *N* = 760.

The nonparametric Spearman correlation was 0.9180 (95% CI 0.9056 to 0.9288) for i-STAT^®^ vs. the LA Plus™ meter. This suggests the correlations for i-STAT^®^ vs. the LA Plus™ meter to be very good. Figure 1 shows the linear regression and its goodness of fit (R^2^), comparing the lactate tests with an R^2^ value of 0.9640. 

### 3.3. Lactate Device Comparison for All Blood Draws

**Figure 2:**
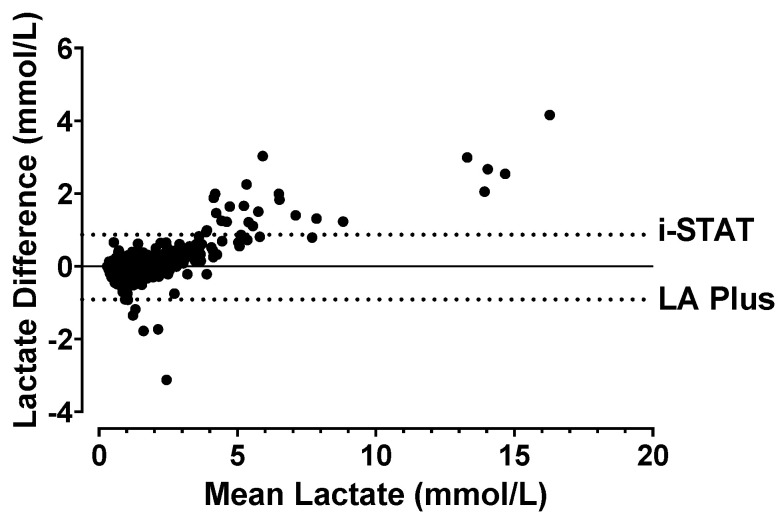
Bland–Altman plot comparing different lactate device values from all the blood samples. This plot is a simple way to evaluate the bias between two quantitative measurements by determining their mean and difference then constructing 95% agreement intervals of bias ± 1.96 standard deviation (dotted lines not at a difference of 0). The negative bias identified indicates that the test on the bottom of the figure typically has larger measurements. A bias closer to 0 signifies the measurements are in agreement and thus the methods are similar.

The Bland–Altman plot of the samples measured on the lactate devices is shown in Figure 2. The comparison of i-STAT^®^ vs. the LA Plus™ meter resulted in a bias of −0.02270 ± 0.4542 with 95% limits of agreement, from −0.09129 to 0.8675. Even though, overall, there was a negative bias between the two tests, implying that the LA Plus™ meter generally results in a higher value, the i-STAT^®^ showed higher values at higher concentrations (>2 mmol/L) of lactate.

## 4. Discussion

This study represents the first direct comparison between the i-STAT^®^ and the Lactate Plus™ meter in a preclinical trauma swine model, mimicking a prehospital setting. The measured lactate levels followed a predictable pattern: they initially increased after trauma and then gradually returned to the baseline, as expected. Even though our results indicate a minimal difference of −0.02 mmol/L between the two POC devices with the i-STAT^®^ resulting in higher values at lactate >2.5 mmol/L, there were no statistically significant differences between the lactate values of the two devices at any time. The observed similarity in performance between the two devices suggests that both are capable of reliably measuring lactate levels in a trauma situation.

Our results are consistent with the existing literature on lactate measurement, although previous studies have primarily focused on comparing lactate levels in whole blood and plasma [4,21,22]. Studies by Tolan et al. and Leguillier et al. showed there was a good correlation between whole-blood lactate levels measured with POC devices and plasma lactate levels [18,23]. Although some studies indicated lower lactate values with POC devices compared to central laboratory analyzers [24,25]. This may be due to the fact that, unlike the POC tests, the laboratory chemistry analysis was conducted with plasma rather than whole blood, possibly concentrating the analyte during the separation of the components.

POC devices play an important role in the assessment and treatment of prehospital trauma patients and should be implemented in regular prehospital algorithms for patient treatment, trauma triage, and risk stratification [24,26,27,28]. The practical advantages of POC devices in prehospital trauma treatment are also demonstrated by the fact that only a small sample volume is required, and results are delivered quickly. Small amounts of whole blood can be used either from a venous access port or through a drop of capillary blood, acquired in a quick and uncomplicated manner from a finger or earlobe, enabling diagnostics with little invasiveness. This is important in patients with hemorrhagic shock and the centralization of blood, where there are difficulties in establishing an access line. The Lactate Plus™ meter requires only about 1 µL of blood and delivers results in 13 s, compared to the i-STAT^®^, with up to 100 µL and a turnaround time of 120 s [4,29]. These features are particularly important in prehospital settings where fast assessment is necessary and real-time decision making in terms of adequate early treatment is required, such as at the scene of an accident or during transportation to the hospital. Serial lactate measurements with POC devices in these settings permit the real-time detection of oxygen deficits, prevent the underestimation of hemorrhagic shock, and can guide resuscitation efforts. This is particularly important in settings where conventional clinical signs of hypoperfusion are difficult to detect [4,5,26,28,30,31,32,33,34,35]. It also enables the assessment of the time course and the timely identification of patients at risk of dying or those in need of a transfusion [36]. This is especially true for patients who cannot actively express their well-being, such as unconscious people or children [32]. In addition, early lactate measurement in mass casualty incidents may help to objectively support triage, prioritize the treatment of patients at high risk of hemorrhagic shock, and thus improve the chances of survival [26]. A study by Lee et al. emphasizes that POC lactate measurements can facilitate early sepsis diagnosis and monitoring in the emergency room [37]. POC devices are very economical as they are small, lightweight, easy to handle, and require little storage space, making them ideal for prehospital use [19,21,26]. In addition, minimal maintenance and calibration efforts are required [4]. Compared to the i-STAT^®^, the LA Plus™ meter is cheaper in terms of acquisition, maintenance, and testing supplies [38]. Further, the testing strips for the LA Plus*™* meter can be stored at 15–30 °C, with an extended shelf life of up to 24 months.

Despite their usefulness, lactate values should be interpreted with caution and as an adjunct to the overall clinical picture. Lactate is a non-specific biomarker that is influenced by a variety of physiological processes. Factors such as the infusion of lactated Ringer’s solution or prolonged tourniquet use can artificially elevate lactate levels, underscoring the need for a comprehensive patient assessment when interpreting results.

This study was subject to several limitations. First, the use of an animal model may not fully reflect the complexity of human physiological responses. Second, therapeutic interventions were not adjusted for lactate levels. Third, the blood samples were partially collected at different sites (e.g., femoral vs. carotid/jugular) with less than <6% being venous, which may have influenced the results. Each blood draw was performed only once per test so an assessment of precision (intra-assay variation) is not possible. The exact timing of sample processing with the POC devices was not recorded, but it was generally within 15 min of blood draw. However, the two POC tests were run simultaneously, so no differences in individual blood draw comparisons were expected. As already stated, values were missing for some time points. Fourth, the lack of comparison with a blood gas analyzer, which is commonly used in clinical practice, limits the applicability of the results on a broad basis. Fifth, the LA Plus™ meter used is not FDA-cleared, although the company offers similar FDA-cleared technology with test strips requiring only a drop of blood. Lastly, the device lacks network or cloud-based capabilities for data transmission. A major strength of this preclinical work is that both male and female swine of two strains were utilized, and no differences were identified.

Future research should focus on the validation of POC devices in human subjects, especially in different clinical scenarios such as trauma and sepsis. Comparative studies between capillary and venous blood sampling would also help to establish standardized protocols for lactate measurement. Increasing the sample size and conducting studies in real-world settings will further improve the generalizability of these results and support the integration of lactate-guided therapies in both prehospital and inpatient care.

## 5. Conclusions

The LA Plus™ meter demonstrated the rapid acquisition of lactate values using only a drop of blood. This device does not require cold-chain storage and has a long shelf life for its testing strips. This study provides a proof of concept that the LA Plus™ meter is comparable to the i-STAT^®^ as a POC device to measure lactate in polytraumatized patients.

## Figures and Tables

**Table 1:** Overall preclinical trauma models included in the observational analysis.

Injury Model	Swine [*n*]	Blood Draws with Both Devices [*n*]
40% Total body surface area burn with bacterial inoculation (Burn)	15	170
Fecal peritonitis (FP)	11	109
Penetrating abdominal trauma (AT)	25	277
Complex extremity trauma injury (CETI)	23	204
TOTAL	74	760

**Table 2:** Combined lactate values over time for both lactate tests.

Time Point	Swine [*n*]	i-STAT^®^ [mmol/L]	LA Plus™ Meter [mmol/L]
Before Injury (BI)	145	1.00 ± 0.52	1.08 ± 0.49
Immediately After Injury (IAI)	70	3.17 ± 2.90 #	2.82 ± 2.30 #
Day 1	225	1.73 ± 2.12 @	1.71 ± 1.68 *
Day 2	152	0.99 ± 0.82	1.09 ± 0.66
Day 3	146	0.99 ± 1.00	1.09 ± 0.73
Day 4	22	0.62 ± 0.26	0.85 ± 0.35

Data are presented as mean ± standard deviation. # = IAI compared to all other time points for that particular lactate test. @ = Day 1 compared to the BI, Day 2, Day 3, and Day 4 time points for the i-STAT^®^ test. * = Day 1 compared to the BI, Day 2, and Day 3 time points for the LA Plus™ meter. All significance was *p* < 0.05.

## Data Availability

Due to the size of the raw files, datasets are available upon request.
